# Evaluating the distribution of the locations of colorectal cancer after appendectomy and cholecystectomy

**DOI:** 10.1186/s12957-020-01861-4

**Published:** 2020-05-12

**Authors:** Szabolcs Ábrahám, Tibor Németh, Ria Benkő, Mária Matuz, Aurél Ottlakán, Dániel Váczi, Attila Paszt, Zsolt Simonka, György Lázár

**Affiliations:** 1 Department of Surgery, Szent-Györgyi Albert Medical and Pharmaceutical Center, University of Szeged, Semmelweis u. 8., Szeged, H-6725 Hungary; 2 Department of Clinical Pharmacy, University of Szeged, Szeged, Hungary

**Keywords:** Tumor localization, Tumor, Appendectomy, Cholecystectomy, Colorectal cancer

## Abstract

**Backgrounds:**

The number of appendectomies and cholecystectomies performed is gradually increasing worldwide. An increasing incidence of colorectal cancer (CRC) after appendectomy and cholecystectomy has been reported, but the location of tumors in certain segments of the colon and rectum after appendectomy and cholecystectomy is still uncertain. We aimed to evaluate the distribution of the locations of colorectal cancer after appendectomy and/or cholecystectomy in patients who underwent CRC surgery.

**Methods:**

We reviewed the medical records of patients who had undergone CRC surgery between 2015 and 2017 for the presence of previous appendectomy/cholecystectomy. Data were collected from the Colorectal Data Base of the University of Szeged, Department of Surgery.

**Results:**

Surgery for CRC was performed in 640 patients during the study period. Data of 604 patients were analyzed. Appendectomy was performed in 100 patients (16.6%), cholecystectomy in 65 (10.8%), and both interventions in 18 (3%) before the CRC surgery. Out of those patients who underwent appendectomy alone, 92 (92%) had undergone appendectomy more than 10 years before the CRC surgery. Also in these 100 patients, the prevalence of right-sided colon cancer (CC) was 35% (*n* = 35), in comparison with the prevalence among the 504 other patients (20.4%, *n* = 103). The prevalence of right-sided CC among patients who underwent cholecystectomy alone was 36.9% (*n* = 24), in comparison with 21.2% (*n* = 114) of the 539 other patients.

**Conclusions:**

A significant left to right side shift in CRC was noted among patients who had previously undergone appendectomy/cholecystectomy. Because right-sided CC has a worse prognosis, the role of incidental appendectomy and routine cholecystectomy seems that need re-evaluation.

## Introduction

Colorectal cancer (CRC) was the third most common malignant tumor diagnosed in 2012 worldwide (1.4 million patients) and the fourth most common cause of death (700,000 patients) [1]. CRC was responsible for approximately 150,000–175,000 deaths annually in the European Union between 2011 and 2018 [2, 3]. The incidence of CRC is expected to increase further. The incidence and rate of mortality due to CRC have increased in most of the countries except in those in which public health measures (such as CRC screening) have been initiated [1].

The prevalence of CRC shows geographical differences and is associated with the degree of industrialization in a country. The risk factors of CRC can be classified as modifiable and non-modifiable. Risk factors related to lifestyle or drug treatment include alcohol consumption, obesity, smoking, red meat consumption, sedentary lifestyle, postmenopausal hormonal treatment, non-steroidal anti-inflammatory drug treatment (100 mg of acetylsalicylic acid daily), and decreased consumption of vegetables and fruits. Non-modifiable risk factors include inflammatory bowel diseases, positive family history, age, and gender [4]. Possible additional risk factors of CRC include a history of appendectomy and cholecystectomy [5, 6].

The average incidence of either appendicitis or appendectomy is 100–206 per 100,000 people [7]. McVay, in 1964, was the first to observe that a significantly high percentage of patients who died of CRC had previously undergone appendectomy [8]. Later, large population cohort studies showed increased prevalence of CRC (up to 14%) after appendectomy [5]. Cholecystectomy was also found to be associated with higher prevalence of CRC [6]. In patients who had previously undergone cholecystectomy, the incidence of CRC was 119 per 100,000, in comparison to that of 86 per 100,000 in patients who had not undergone cholecystectomy [9].

With regard to the location of CRC, the left side of the colon is the common site in most patient population [5, 10, 11]. The location of tumors in certain segments of the colon and rectum after appendectomy and cholecystectomy is still uncertain [5, 12, 13]. Wu et al. reported that not only did patients who underwent appendectomy have an increased incidence of CRC but also the CRC location shifted [5], with increased tumor development in the rectum.

As the prognosis and survival rate of right-side CC is convincingly proved to be worse than left side CRC, the location of the tumor development is of utmost importance [14–17].

We aimed to evaluate the distribution of the locations of colorectal cancer after appendectomy and/or cholecystectomy in patients who underwent CRC surgery. We did not aim to analyze the causal relationship between appendectomy/cholecystectomy and development of colon cancer.

## Patients and methods

The study was conducted with the ethical approval of the Ethics Committee of the University of Szeged (190/2015-SZTE) and the Medical Research Council (TUKEB 2655-2/2018/EKU). We gathered data from the Colorectal Data Base of the University of Szeged Department of Surgery and from the e-MedSolution® electronic integrated hospital system.

The medical histories of patients undergoing CRC surgery between 2015 and 2017 were analyzed. We sought a possible association between the location of CRCs and previous appendectomy/cholecystectomy. Patients were classified into four groups: Those in the CRC group had not previously undergone appendectomy or cholecystectomy but did have surgery for CRC, those in the CRC + APP group had previously undergone appendectomy and later surgery for CRC, those in CRC + CCY group had undergone cholecystectomy and later surgery for CRC, and those in the CRC + APP + CCY group had undergone previous appendectomy and previous cholecystectomy and later surgery for CRC.

We conducted univariate analysis (chi-square test) to analyze the associations among different patient characteristics, CRC predisposing factors (gender, age, smoking, alcohol consumption, acetylsalicylic acid treatment (100 mg per day), history of previous CRC, family history, previous appendectomy, and cholecystectomy), and tumor main location (left colon, right colon, rectum).

**Fig. 1:**
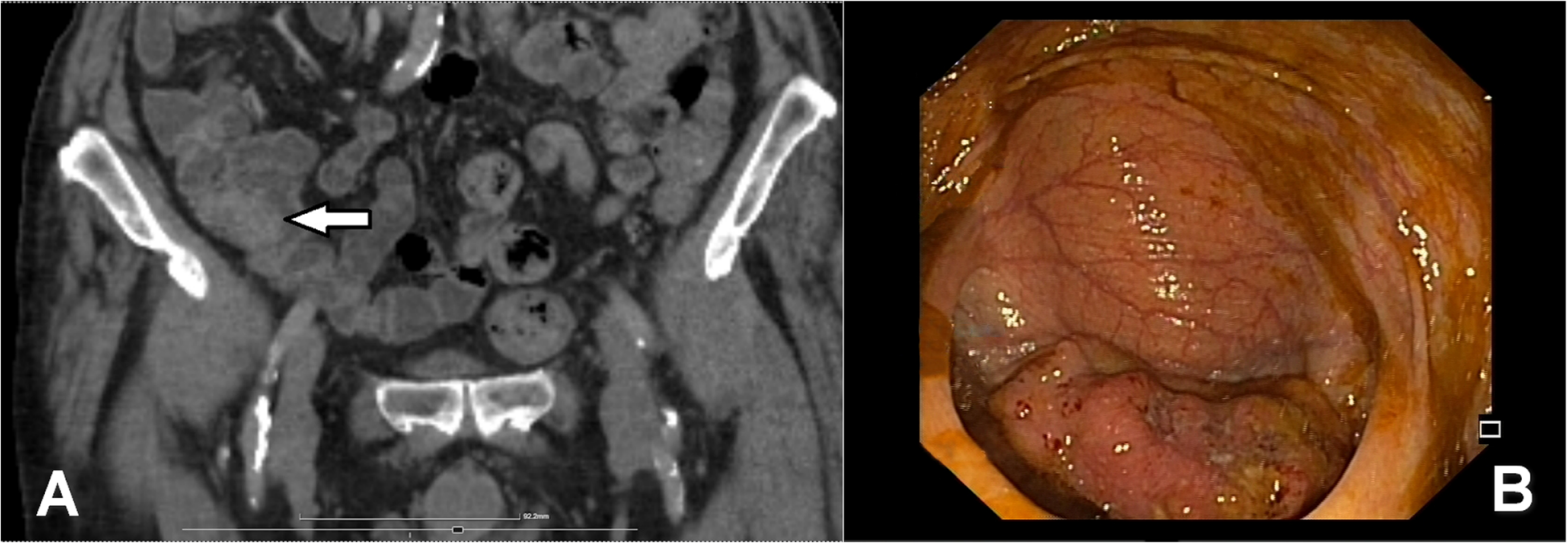
**a**, **b** Abdominal computer tomography (CT) and colonoscopic image of cecal cancer

The detailed distribution of CRC location (in certain colon segments) was also analyzed. Right-sided CCs were located in the caecum, ascending colon, hepatic flexure, and transverse colon, whereas left side CRCs were located the left colon segment (splenic flexure, descending colon, and sigmoid colon). Tumor location was determined by colonoscopic and abdominal computer tomography (CT) results (Fig. 1a, b).

## Results

A total of 640 patients underwent surgery due to CRC. Thirty-six patients were excluded from the study (the history of appendectomy or cholecystectomy was unclear in 9 patients, bilateral synchronous tumor was present in 24 patients, and exact tumor location was unclear in 3). Data from the remaining 604 patients were statistically analyzed.

### General characteristics of patients

Of the patients, 350 (57.9%) were male; mean age was 66.5 years (standard deviation ±11.47 years; range, 21 to 97 years), and 336 patients (55.6%) were older than 65 years. The majority of patients did not smoke, and alcohol consumption was generally not a risk factor: 526 patients (87.1%) had never smoked, and 78 (12.9%) were current or former smokers. Alcohol consumption was excessive in 24 patients (4.0%), and 90 patients (14.9%) were taking products containing acetylsalicylic acid. Colorectal tumor was documented in the past medical history of 20 patients (3.3%) and in the family history of 19 (3.1%). One hundred patients (16.6%) had previously undergone appendectomy, 65 (10.8%) had previously undergone cholecystectomy, and 18 (3%) had undergone both procedures.

### Colorectal cancer localization

Tumor location was rectal in 259 patients (42.9%), left-sided in 207 (34.3%), and right-sided in 138 (22.8%) of cases. Detailed examination of locations revealed that 54 patients (8.9%) had tumors of the caecum, 29 (4.8%) had tumors of the ascending colon, and 26 (4.3%) had tumors of the hepatic flexure. In addition, 29 (4.8%) had transverse colon tumors, 14 (2.3%) had splenic flexure tumors, 17 (2.8%) had tumors of the descending colon, 176 (29.1%) had tumors in the sigmoid and rectosigmoid junction, and 259 (42.9%) had rectal tumors (Table 1).

**Table 1:** General data of enrolled patients

Characteristic	Number	Percentage
Gender		
Male	350	57.9
Female	254	42.1
Age		
Mean ± SD	66.46 ± 11.47 years	
Range	21–97 years	
Above the age of 65 years	336	55.6
**S**moking		
Smoking	52	8.6
Quit smoking	26	4.3
Not smoking	526	87.1
Alcohol consumption	24	4.0
Acetylsalicylic therapy	90	14.9
Previous CRC	20	3.3
Family history	19	3.1
APP		
In the past medical history	100	16.6
More than 10 years earlier	92	15.2
CCK		
CCK in the past medical history	65	10.8
APP and CCK together	18	3.0
Location		
Left side of the colon	207	34.3
Right side of the colon	138	22.8
Rectum	259	42.9
Specific location		
Caecum	54	8.9
Ascending colon	29	4.8
Hepatic flexure	26	4.3
Transverse colon	29	4.8
Splenic flexure	14	2.3
Descending colon	17	2.8
Sigmoid and rectosigmoid colon	176	29.1
Rectum	259	42.9

Total *N* = 604 patients

*APP* appendectomy, *CCK* cholecystectomy, *CRC* colorectal cancer, *SD* standard deviation

### Association between tumor location and different patient factors

The findings of the associations between different patient factors and main tumor location are summarized in Table 2. CRC localization had similar distribution in the left and right colon and also the rectum among both genders (male 35.1%; 20.0% and 44.9% vs female 33.1%; 26.8% and 40.2%). Those with right-sided CC were older, although age-group analysis has not revealed any statistically significant difference in the tumor localization between the two age-groups (under and above 65 years). Tumor localization did not differ in those with different lifestyle habits (smoking, alcohol consumption) or medication history with low-dose acetylsalicylic acid. Previous CRC in the medical or family history was also not associated with different distribution of CRC localization (Table 2).

**Table 2:** Evaluation of the tumor location with univariate method

		Left	Right	Rectum	Ʃ	Test, *p* value
Total		207	138	259	**604 (100%)**	
Gender	Male (%)	123 (35.1%)	70 (20%)	157 (44.9%)	**350 (100%)**	Pearson’s chi-squared test; *p* = 0.1425
	Female (%)	84 (33.1%)	68 (26.8%)	102 (40.2%)	**254 (100%)**	
Age	Mean ± SD	66.3 ± 11.4	70.1 ± 11.3	64.6 ± 11.2		ANOVA, *p* < 0.0001 Tukey’s multiple comparisons of means: left-right *p* = 0.0068829; rectum-right *p* = 0.0000151; rectum-left *p* = 0.2394
	Min–max	28–96	41–97	21–91		
	Above the age of 65 (%)	112 (33.3%)	96 (28.6%)	128 (38.1%)	**336 (100%)**	Pearson’s chi-squared test; *p* value = 0.1736
	Maximum 65 years old	95 (35.4%)	42 (15.7%)	131 (48.9%)	**268 (100%)**	
Smoking	Smoking	14 (26.9%)	7 (13.5%)	31 (59.6%)	**52 (100%)**	Pearson’s chi-squared test; *p* = 0.05634
	Quit smoking	5 (19.2%)	5 (19.2%)	16 (61.5%)	**26 (100%)**	
	Not smoking	188 (35.7%)	126 (24%)	212 (40.3%)	**526 (100%)**	
Alcohol consumption	Regular alcohol consumption	4 (16.7%)	6 (25%)	14 (58.3%)	**24 (100%)**	Pearson’s chi-squared test; *p* = 0.157
	No alcohol consumption	203 (35%)	132 (22.8%)	245 (42.2%)	**580 (100%)**	
Acetylsalicylic therapy	Acetylsalicylic acid intake	25 (27.8%)	26 (28.9%)	39 (43.3%)	**90 (100%)**	Pearson’s chi-squared test; *p* = 0.22306
	No acetylsalicylic acid intake	182 (35.4%)	112 (21.8%)	220 (42.8%)	**514 (100%)**	
Previous CRC	Positive	6 (30%)	4 (20%)	10 (50%)	**20 (100%)**	Pearson’s chi-squared test; *p* = 0.8073
	Negative	201 (34.4%)	134 (22.9%)	249 (42.6%)	**584 (100%)**	
Family history	Positive	5 (26.3%)	2 (10.5%)	12 (63.2%)	**19 (100%)**	Pearson’s chi-squared test; *p* = 0.1698
	Negative	202 (34.5%)	136 (23.2%)	247 (42.2%)	**585 (100%)**	
APP	Previous APP	29 (29%)	35 (35%)	36 (36%)	**100 (100%)**	Pearson’s chi-squared test; *p* = 0.006601
	No previous APP	178 (35.3%)	103 (20.4%)	223 (44.2%)	**504 (100%)**	
	*APP performed more than 10 years ago*	*28 (30.4%)*	*32 (34.8%)*	*32 (34.8%)*	***92 (100%)***	
CCK	Previous CCK	16 (24.6%)	24 (36.9%)	25 (38.5%)	**65 (100%)**	Pearson’s chi-squared test; *p* = 0.01337
	No previous CCK	191 (35.4%)	114 (21.2%)	234 (43.4%)	**539 (100%)**	

*CRC* colorectal cancer, *APP* appendectomy, *CCK* cholecystectomy

The history of cholecystectomy or appendectomy in the medical history was associated with remarkable and statistically significant differences in tumor localization: the incidence of right-sided CC was 35% among patients who had previously undergone appendectomy, in comparison with 20.4% among those who had not. The incidence of right-sided CC was 36.9% among patients who had previously undergone cholecystectomy, in comparison with 21.2% among those who had not.

### Distribution of CRC locations in different segments of the colon and rectum

**Fig. 2:**
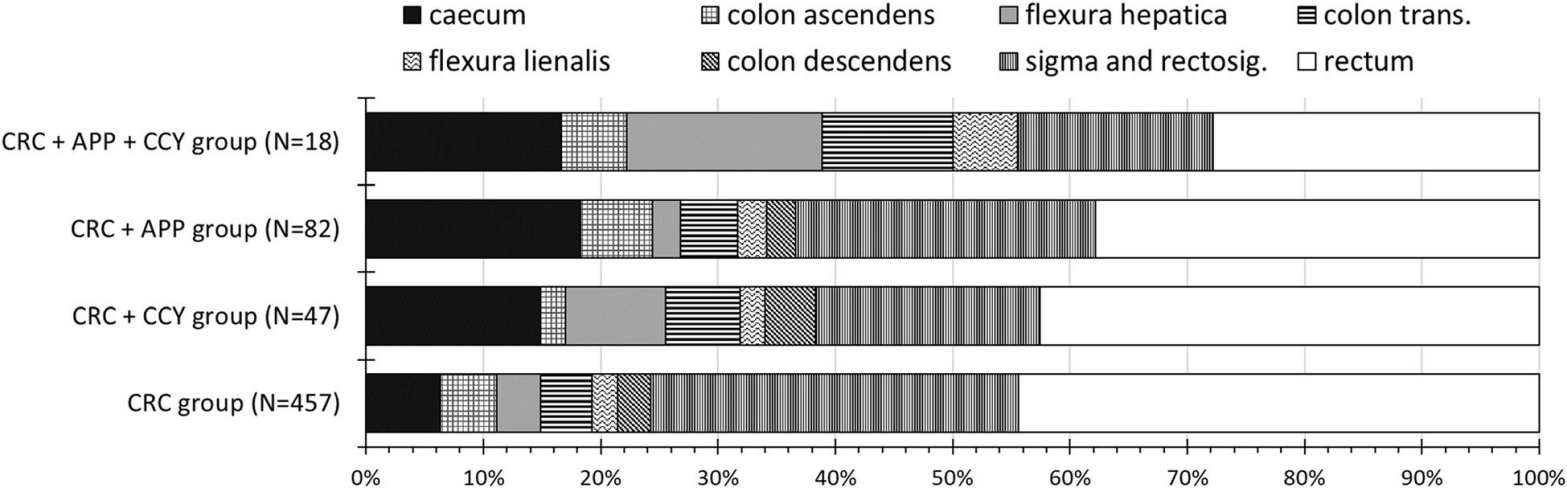
Distribution of colorectal cancers in various locations. CRC, colorectal cancer surgery without previous appendectomy or cholecystectomy; CRC + CCY, colorectal cancer surgery with previous cholecystectomy; CRC + APP, colorectal cancer surgery with previous appendectomy; CRC + APP + CCY, colorectal cancer surgery with previous appendectomy and cholecystectomy

Figure 2 shows the segmental distribution of CRC locations. Overall, right-sided CC was present in 88 patients (19.3%) in the CRC group, 17 patients (31.9%) in the CRC + CCY group, 26 patients (31.7%) in the CRC + APP group, and 9 patients (50%) in the CRC + APP + CCY group. Among right-sided CCs, cecal cancers showed remarkable variations across among different patient groups: cecal cancer was present in 29 patients (6.3%) in the CRC group, 7 patients (14.9%) in the CRC + CCY group, 15 patients (18.3%) in the CRC + APP group, and 3 patients (16.7%) in the CRC + APP + CCY group. We observed an inverse tendency in the distribution of rectal cancers: we found the highest rates among those without previous appendectomy and cholecystectomy (Fig. 2).

## Discussion

There are few international publications focusing on the location distribution of CRCs after gastrointestinal surgical interventions [5, 18]. In most published studies, the investigators examined the relationship between appendectomy or cholecystectomy and CRC development, with differences in CRC incidence [6, 9, 19, 20]. After such operations, the shift in the locations of CRCs may suggest some ground of causation for these procedures.

Our results clearly confirm the predominance of right-sided CCs (i.e., an obvious left to right shift of CRC location) in those patients who have previously undergone appendectomy or cholecystectomy. According to literature, rectal and sigmoid CRCs are the most frequently seen entities, which fact is also consistent with our own results [14]. In addition to the African-American population carrying a 1.23 relative risk of developing right-sided CC, in comparison to the Caucasian population, the National Cancer Institute Surveillance, Epidemiology, and End Results database in the USA showed that right-sided CCs are more prevalent in elderly patients and women. Our results are consistent with this statement. The incidence of CRC proved to be higher in women and in patients older than 65 years, than in men and those younger than 65 years, respectively, although these differences were not statistically significant. Although the overall incidence of CRCs has decreased in the USA, the prevalence of right-sided CCs annually increases by 0.68%, with nearly a 25.26% increase in the past 30 years [21]. Due to differences in the embryological origins of the two sides of the colon, the microbiota, histological appearance, and genetic/molecular features of right- and left-sided colon cancers differ significantly; thus, prognosis for left- and right-sided CCs varies [11, 22]. Right-sided CC is usually diagnosed at a more advanced local stage; metastases are more likely to be present in lymph nodes showing higher tumor grade, which the results in poor prognosis and survival rate [14–17].

In terms of the tumors of the colon and rectum, the shift of incidence from the left to the right side seems contradictory [5, 12]. The predominance of right-sided CC—especially in the caecum—was also confirmed by our study; thus, pathogenesis of CRCs may differ depending on location.

Furthermore, the effects of both previous procedures (appendectomy and cholecystectomy) may be synergistic. Factors that may potentially influence the pathogenesis of cancers after appendectomy include loss of immune function (e.g., microbiota-biofilm alterations) that can weaken the “safe house” role of the appendix [5, 23–25]. Furthermore, elapsed time since appendectomy can also play a role in tumor development. The most often emphasized factors after cholecystectomy include increased secretion of hydrophobic bile acid into the colon, dysbiosis of the microbiota, increased permeability of the colonic epithelium, increased bacterial translocation, and altered transmembrane and intracellular cascade mechanisms that influence apoptotic resistance of cells [26].

In most relevant studies, investigators have evaluated the incidence of CRCs and their location only after either appendectomy or cholecystectomy. Our study focuses on the effect of both interventions, both individually and with one another. Although the overall incidence of post-cholecystectomy CRC has significantly increased in recent years, this phenomenon has not yet been proven for rectal tumors [27].

During a 5–15-year follow-up period after appendectomy, Song et al. identified appendectomy as a risk factor in the development of right-sided CC [13]. In a retrospective study, Lee et al. found that the incidence of colon cancer (5.74%) increased significantly during the first year after appendectomy in comparison with rectal cancer (1.89%) [12]. In a prospective study by Wu et al., an increased incidence of CRC was seen during a 1.5–3.5-year follow-up period after appendectomy (with slightly increased incidence of rectal cancer) [5].

In their study including 707,663 patients, Lee et al. reported that the incidence of CRC significantly increased during the first 3 years after appendectomy, and the same phenomenon was observed 1 year after cholecystectomy [12]. In terms of elapsed time, we found that in 91.8% of patients, a more than 10-year long time period was seen between the initial appendectomy and the development of CRC.

In the abovementioned studies, the investigators focused on the incidence of CRC after certain surgical procedures, whereas our aim was to analyze the differences in the distribution of tumor location. Our results raise further questions regarding the effects of incidental appendectomy. Incidental or prophylactic appendectomy is an intervention during which an otherwise macroscopically intact appendix is removed. It is often performed during gynecological procedures and less commonly during surgical or urological interventions. Different authors have found the rate of appendectomies without appendicitis to be between 3.6 and 47%, with doubled incidence in women [28, 29]. These results raise the question whether the number of appendectomies should be decreased both by avoiding incidental appendectomies and by forcing conservative treatment for acute uncomplicated appendicitis [30].

The indications of cholecystectomies should be narrowed down: cholecystectomy can be avoided in asymptomatic, accidentally diagnosed cases with cholecystolithiasis. Furthermore, with the assumption of a possible relationship between right-sided CC and a history of previous appendectomy or cholecystectomy, indications for incidental appendectomies and cholecystectomies should be re-evaluated. The former suggestion would require further epidemiological investigations.

Our study also had some limitations. First, our analysis focused only on operated CRC cases and not on all diagnosed CRC cases. Secondly, since a single-center study, its findings cannot be directly applied to the general patient population. Thirdly, since not all CRC-associated factors (e.g., dietary factors, mental state, obesity) were assessed, this may also influence CRC location.

## Conclusions

With regard to the location of CRCs, our results indicate the predominance of the right-sided colon segment (mainly the caecum) after appendectomy and cholecystectomy. The importance of the above mentioned observation is that the prognosis of right-sided CCs is worse than that of the left colon side. The indication of appendectomy and routine cholecystectomy should be considered more carefully. Further studies with control group (non-CRC) are needed to evaluate the role of appendectomy and cholecystectomy in CRCs.

## Data Availability

The datasets generated and/or analyzed during the current study are not publicly available because of the data protection of the database, but are available from the corresponding author on reasonable request.

## Abbreviations

CRC: Colorectal cancer
CC: Colon cancer
APP: Appendectomy
CCK: Cholecystectomy
SD: Standard deviation
